# Genetic discrimination by Australian insurance companies: a survey of consumer experiences

**DOI:** 10.1038/s41431-019-0426-1

**Published:** 2019-07-08

**Authors:** Jane Tiller, Susan Morris, Toni Rice, Krystal Barter, Moeen Riaz, Louise Keogh, Martin B. Delatycki, Margaret Otlowski, Paul Lacaze

**Affiliations:** 1 Public Health Genomics, School of Public Health and Preventive Medicine, Monash University, Melbourne, VIC Australia; 2 Lynch Syndrome Australia, The Summit, QLD Australia; 3 Pink Hope Community Limited, Narrabeen, NSW Australia; 4 Centre for Health Equity, Melbourne School of Population and Global Health, The University of Melbourne, Melbourne, VIC Australia; 5 Victorian Clinical Genetics Services, Parkville, VIC Australia; 6 Murdoch Children’s Research Institute, Parkville, VIC Australia; 7 Centre for Law and Genetics, Faculty of Law, University of Tasmania, Hobart, TAS Australia

**Keywords:** Cancer genetics, Ethics, Medical ethics

## Abstract

We report previously undocumented evidence of genetic discrimination by Australian insurance companies, obtained through direct consumer reports. We surveyed 174 consumers with cancer-predisposing variants, recruited by cancer organisations Lynch Syndrome Australia and Pink Hope. Questions related to experiences accessing risk-rated insurance after genetic testing. Results indicate that both legal (permitted under current regulation) and illegal discrimination is occurring. Although some respondents had not applied for risk-rated insurance, or had insurance in place before genetic testing (*n* = 100), those seeking new policies (*n* = 74) commonly experienced difficulties obtaining insurance (86%, 64/74). Of those experiencing difficulties, 50% (32/64) had no prior history or symptoms of cancer, and had undertaken risk reduction through surveillance and/or preventative surgery. Seventy-seven percent (49/64) reported difficulties related to life insurance. Follow-up telephone interviews with four respondents further described cases of apparent illegal breaches. All reports of discrimination identified were, to our knowledge, previously unreported in the literature. The number of cases suggests a systemic problem with the Australian life insurance industry. We support calls for government oversight of the inherently conflicted model of industry self-regulation in Australia, and an immediate ban on the use of genetic test results in insurance underwriting.

## Introduction

In Australia, discrimination on the basis of genetic status is prohibited by the *Disability Discrimination Act 1992*(Cth), but an exception allows insurance discrimination on actuarial grounds. Insurers can use genetic test results, even in the absence of disease symptoms, to deny coverage or impose increased premiums on products such as life, income protection, permanent disability and travel insurance. However, genetic discrimination must be actuarially or reasonably justified. Insurers must consider risk-reducing measures, including surveillance and surgery. This requirement creates a distinction between *legal* genetic discrimination, where an insurer’s actions are within current legal regulation (but ethically contentious [1]), and *illegal* genetic discrimination—where an insurer’s behaviour is in breach of a clear regulatory requirement. Private health insurance, which is community rated in Australia (no individual risk assessment), is not affected.

Predictive genetic testing can identify modifiable disease risk, especially for cancer, where life-saving intervention is possible. Conditions commonly benefiting from predictive testing include Hereditary Breast and Ovarian Cancer syndrome (*BRCA1/2* genes) and Lynch syndrome, which increases risk for colon, endometrial and other cancers. Risk-reduction programs and preventive surgeries are available and highly effective for both of these conditions. Preventative surgery in *BRCA1/2* carriers (mastectomy/salpingo-oophorectomy) can reduce risk of breast and/or ovarian cancer to below population average [2]. Intensive breast surveillance also reduces risk, with 10-year survival >95% with magnetic resonance imaging screening [3]. For Lynch syndrome, surveillance through colonoscopy and chemoprevention significantly reduces colorectal cancer incidence and mortality [4], and preventative gynaecological surgery significantly reduces risk of gynaecological cancer [5].

Previous studies have shown that life insurance companies in Australia do not always consider risk reduction in underwriting [6–8]. In addition, several consumers have recently come forward to the media, reporting difficulties accessing life insurance, even after undergoing risk-reducing surgery and/or surveillance. In one well-documented case, involving an application to the Australian Human Rights Commission, multiple life insurers failed to consider risk reduction for Lynch syndrome when underwriting [7].

Further evidence indicates consumer fears regarding insurance deter the uptake of genetic testing and participation in medical research in Australia [9–12]. In one study, individuals informed of possible insurance implications of genetic testing were more than twice as likely to decline testing as those not informed [10]. This is an acknowledged barrier to progress in genomic medicine, compromising Australian medical research [13]. Further, the current model of industry self-regulation has resulted in inadequate transparency and limited accountability for policy breaches [14].

In March 2018, a Parliamentary Joint Committee (PJC) inquiry into the Australian life insurance industry released its official report [13], recommending an urgent ban on the use of predictive genetic test results. The PJC recommended a ban similar to the UK moratorium, and informed by bans in other countries [14, 15]. To date, no ban has been implemented in Australia. The Financial Services Council (FSC) recently announced an industry-regulated moratorium to commence mid-2019, which falls short of the PJC’s recommendations—it has significant financial limits and no government involvement. At the time of writing, the FSC has commenced consultation on the proposed terms.

Historically, it has been difficult to quantify the scale of genetic discrimination in Australian insurance, and identify new cases. Accessing records of underwriting decisions by life insurers has been difficult, and controlled by the peak insurer body, the FSC. Data on underwriting has been periodically made available to researchers for analysis and resulting publication [8, 16]. However, published datasets have typically lacked completeness, with limited numbers of insurers contributing data, and have been significantly out-of-date by publication.

This study aimed to identify previously unreported cases of genetic discrimination in Australian insurance, through direct consumer reports.

## Materials and methods

We partnered with education and advocacy bodies for individuals with inherited cancer-predisposing variants: Lynch Syndrome Australia (LSA) and Pink Hope (PH). These organisations have broad networks of consumers undertaking genetic testing. As there is no national register of variant carriers in Australia, these support organisations provided the most efficient and direct access to a convenience sample of consumers who may have experienced difficulties accessing insurance products after genetic testing.

Our sequential mixed-methods study was descriptive and exploratory. It comprised an online survey designed to identify experiences of discrimination on the basis of genetic test results by insurance companies, followed by targeted telephone interviews to describe selected consumer experiences in greater detail. Products offered by life insurance companies (including life, income protection and disability insurance, collectively referred to hereafter as “life insurance products”) were of primary interest in this study. However, we also included reports of discrimination in other risk-rated insurance, including mortgage protection and travel insurance. Health insurance, which is community rated, was excluded.

The first survey draft was developed collaboratively with LSA, and then adapted for PH members. While covering the same topics, the surveys were modified slightly between the groups to better capture relevant information. Questions included demographics, relevant medical details, variant detected, prior cancer diagnoses, relatives affected, risk-reducing surveillance/surgery, and insurance experiences (Supplementary Materials). At the end of the survey, respondents were asked whether they would be willing to be re-contacted by researchers to discuss experiences further. Follow-up telephone interviews (semi-structured) were conducted with a limited number of respondents, and were designed to gather more in-depth data on consumer reports of genetic discrimination, particularly related to suspected breaches of current policy.

Surveys were distributed by email and social media to LSA and PH group members, to reach a convenience sample of consumers likely to have experienced insurance discrimination after genetic testing. LSA sent one email and promoted on social media once. PH promoted the survey on social media twice. Semi-structured follow-up telephone interviews were designed to collect more detail from a small subset of the respondents who consented to re-contact. Follow-up respondents were selected on the basis of the type of discrimination reported in the survey. We prioritised respondents who reported what appeared to be illegal discrimination, characterised by a failure of insurance companies to consider risk-reducing surgery. Our aim was not to reach saturation but to gather more data on specific cases of illegal discrimination.

Data analysis involved descriptive statistics of survey responses. Follow-up interviews were recorded and analysed to describe the key features of respondents’ experiences. The project was approved by the Alfred Human Research Ethics Committee, number 332/18.

## Results

LSA emailed its survey to 550 members, then promoted via social media with an estimated reach of 700–800 people, based on metrics reports (September–October 2017). PH promoted its survey via social media in March and April 2018 (estimated reach of 2540). Due to the online recruitment method, overall survey response rates could not be calculated.

We received completed surveys from 214 respondents. Respondents without cancer-predisposing variants and those from outside Australia were excluded. The resulting 174 respondents comprised the final data set (summary results Table 1). The majority were female (91%), and aged 40–60 years (55%). The most common variant was *BRCA1* (23%).

**Table 1:** Summary results from consumer surveys

	Total	PH	LSA
Total survey respondents, *N* = 174			
All	174	74	100
Gender (female)	158	74	84
Age (years)			
Under 40	61	38	23
40–60	95	32	63
Over 60	18	4	14
Genetic risk variant			
* MLH1*	25	–	25
* MSH2*	27	–	27
* MSH6*	16	–	16
* PMS2*	12	–	12
Other/unsure	22	2	20
* BRCA1*	41	41	–
* BRCA2*	31	31	–
Willing to discuss experiences by phone	71	45	26
Difficulty accessing life insurance product	29	7	22
Took part in follow-up interview	4	2	2
Reported difficulty accessing insurance, *N* = 64			
All	64	18	46
Type (multiple possible per respondent)			
Life insurance products Total	49	12	37
Life insurance	39	9	30
Income protection insurance	31	6	25
Disability insurance	20	5	15
Mortgage insurance	11	1	10
Travel insurance	8	8	–
Personal history of cancer	32	10	22
No personal history of cancer, in high-risk surveillance and/or had preventive surgery^a^	32	8	24
Appealed insurer’s decision	6	2	4
Did not report appealing insurer’s decision	58	16	42
Did not know appeal was an option	–	–	21
Other/did not answer question	**–**		21
Did not report difficulty accessing insurance, *N* = 110			
All	110	56	54
Obtained *life insurance* at standard rates after genetic testing	10	3	7
Obtained *travel insurance* at standard rates after genetic testing	13	13	***–***
Already had insurance prior to testing	61	30	31
Did not attempt to obtain insurance/did not answer the question	39	24	15

Dashed fields (–) indicate questions not asked or not relevant

^a^Possible breaches of the industry code and Disability Discrimination Act 1992 (Cth)

Many respondents who obtained insurance did so before genetic testing (55%, 61/110). Another 22% (39/110) reported no attempt to obtain insurance. Of the remainder (*n* = 74), 86% (64/74) experienced difficulty obtaining risk-rated insurance after having genetic testing (Table 1). This included denials of cover (*n* = 45) or premiums increased above standard rates (*n* = 16), with three participants not disclosing the type of difficulty. Of these, 77% (49/64) experienced difficulty obtaining life insurance products.

Of those who reported difficulties, 50% (32/64) had no personal cancer history *and* reported risk reduction (regular surveillance and/or preventative surgery). These represented possible cases of illegal discrimination (for details see Supplementary Table S1). Only 9% (6/64) of respondents who had difficulty reported appealing the insurer’s decision. LSA participants were asked specific questions about reasons for not appealing, and of those who did not appeal the insurer’s decision, 50% (21/42) said they did not know that this was an option (Table S1).

Of the PH respondents without preventative surgery (*n* = 17), all but one were in high-risk breast surveillance. All LSA respondents without bowel cancer (*n* = 57) had regular colonoscopy. Of those who obtained life insurance products after testing (*n* = 10), none had a prior history of cancer, and all had some preventative surgery and/or regular surveillance.

Seventy-one respondents agreed to re-contact for follow-up, 29 of whom had reported difficulty accessing life insurance products. Resource limitations precluded interviewing all 29 respondents. Four respondents were chosen for interviews, whose reported circumstances indicated that risk-reducing measures had not been considered by life insurance companies in underwriting. All four case studies demonstrated aspects of failure by insurers to consider risk reduction. Due to space constraints, and the overlap in issues raised in the four case studies, three were selected to present the experiences of this group (Table 2). We sought pro-bono legal advice regarding Case 1 and Case 3, as they took place in 2018 and were considered the most suitable for legal challenge. We received an initial indication that they could both have grounds for legal challenge on the basis that the insurer was reported to have failed to consider risk reduction in making an adverse decision.

**Table 2:** Case studies

Name^a^ and situation	History of cancer	Risk reduction	Life insurance applications	Appeals	Summary
*Case 1: Evelyn* 47 y/o *BRCA1* variant detected at age 41	No	Bilateral preventative mastectomy and oophorectomy	Financial advisor unable to secure any cover in 2018. “They tried everywhere, but as soon as they mentioned BRCA1, they could not get me any insurance”	Yes Appeal made by financial advisor, unsuccessful “They went back to them and said, ‘look, she’s got no breast tissue, she’s got no ovaries’, but apparently it didn’t matter”	• After receiving positive genetic test results and risk information, Evelyn undertook elective preventative double mastectomy and removal of ovaries/fallopian tubes • In early 2018, after completing surgeries, Evelyn met with a financial advisor and applied for life, income protection and disability insurance. All applications were rejected • Evelyn’s financial advisor appealed the decisions, without success. No actuarial justification was provided
*Case 2: Melanie* 31 y/o Lynch syndrome mutation detected in early 20s	No	Annual colonoscopy and bi-annual gastroscopy; prophylactic gynaecological surgery not recommended until late 30s	Financial adviser advised application would be rejected	No. “Once they heard that I had the gene, they said they’ve tried with other people with that sort of thing and it doesn’t usually get approved, so it wasn’t worth bothering”	• Melanie commenced surveillance for Lynch syndrome after a positive genetic test result in her early 20s • She undertakes annual colonoscopies, bi-annual gastroscopies, as recommended by her specialist, and is considering hysterectomy (recommended at a later age) • Melanie applied for life insurance through a financial advisor, but was advised her application was rejected because of her “condition”, despite no cancer symptoms
*Case 3: Faith* 46 y/o Lynch syndrome mutation detected in early 30s	No	Hysterectomy, oophorectomy, annual colonoscopy, endoscopy	Underwriter at superannuation provider advised that a loading of 100% would be applied to cover (2018)	No Currently considering options	• On specialist advice, Faith undertakes regular surveillance (annual colonoscopy/endoscopy) and had a hysterectomy and oophorectomy • In 2018, Faith applied for increased cover for life, disability and income protection insurance through her superannuation She advised the underwriter of her risk reduction • She was informed that her genetic status would mean loading of the premium by 100%, making her life insurance cover twice as expensive as standard cover

^a^Names changed for confidentiality

## Discussion

Of 64 individuals reporting genetic discrimination by insurers, we identified 32 individuals reporting experiences of arguably illegal genetic discrimination. Based on initial consideration, legal remedies could be pursued in several of these cases.

Difficulties accessing insurance after genetic testing were common in the surveyed population. Strikingly, half of all respondents reporting difficulties accessing insurance had no personal cancer history and were following recommended guidelines for cancer prevention, often reducing risk below population average [2–4], yet they still had policies denied and premiums loaded, without actuarial justification. The limited knowledge of the appeals process reinforces earlier research regarding barriers to uptake of legal remedies [17].

Although some respondents did not report difficulty obtaining insurance, very few actually *obtained* life insurance products at standard rates after receiving a positive genetic test result. Many reported having some insurance in place before having testing, consistent with common pre-test genetic counselling discussions in Australia, regarding considering life insurance before having genetic testing [18].

Discriminating on genetic test results penalises individuals who are proactive about health. By contrast, those who remain uninformed of their risk are not penalised in the same way. Almost all respondents in this cohort took significant steps to reduce their risks of cancer after receiving positive genetic test results. This suggests at-risk consumers who are members of organisations like PH and LSA are motivated to reduce risk, rather than prioritise seeking higher levels of life insurance, although participants were not directly asked this question. This is consistent with a US study showing women with *BRCA1/2* variants did not purchase more life insurance than untested women [19]. This would need to be tested in a larger sample to confirm whether this holds at the population level.

In many cases, individuals bear the costs of proactive risk mitigation, but can still be financially disadvantaged by discriminatory insurance underwriting. Although insurers are legally obliged to consider risk reduction, this study indicates that in many cases, they do not. This means that not only are individuals choosing not to have predictive testing, but further, their subsequent lack of access to preventative measures has potential negative effects on their health and life expectancy. If insurers were to accept a ban as recommended, it would encourage at-risk individuals to take steps to mitigate their risk, making claims less likely [1].

### Legal and illegal discrimination

These and other social policy reasons support an argument that even the *legal* genetic discrimination identified should not be allowed [1]. A ban on the use of genetic test results by insurers, as applied in many other countries and recommended by the Australian PJC [13], would protect consumers from all genetic discrimination in life insurance and allow individuals to pursue genetic testing and participate in research without insurance fears. It is important to note that the recommended ban would not apply to family history information, only genetic test results. Further, the recommended ban would allow the use of genetic test results to counter a negative family history (i.e. to show that an individual does *not* have a genetic variant that runs in the family and contributes to a family history of disease).

In at least 50% of the cases of discrimination reported, insurers failed to consider risk-reducing measures, consistent with illegal genetic discrimination. If substantiated, these cases would indicate insurer disregard for existing regulations, and minimal accountability for breaches of current regulatory requirements. Government oversight and stricter penalties are required to ensure compliance within a self-regulated industry. Legal remedies for insurance discrimination flow from federal and state anti-discrimination legislation [17]. However, the appeal pathway is unclear and the cost of legal advice can be prohibitive, meaning available legal remedies are often not pursued by affected consumers [7, 17]. In our study, only 9% of affected consumers appealed adverse decisions.

### Limitations

Our survey relied on consumer self-reports, and investigators did not have access to primary insurance documentation to verify reports of discrimination. Differences between the LSA/PH surveys resulted in some differences in data collected, and the total reach of the survey could not be calculated accurately due to online advertising methods. We provided our best estimate, based on reported social media statistics. Recruitment through support networks may have resulted in participant bias towards proactive individuals. While this bias is acknowledged and may limit the generalisability of some results, the number of discrimination cases identified remains pertinent. We accept that the sample is likely more educated, computer savvy and resourceful than the general population, and more likely to be aware of and report genetic discrimination. In our view, the extent of discrimination experienced by those without those characteristics (who are not even aware that discrimination is occurring) is underestimated by this study.

This study provides an up-to-date description of reported cases of genetic discrimination in Australian risk-rated insurance, with a particular focus on life insurance. We reached only a fraction of possibly affected consumers, likely underestimating the scale of the problem and future research could survey a broader range of consumers. Our findings highlight the material impact on consumers experiencing both legal and illegal discrimination by Australian insurers. In light of the documented adverse impact of insurance concerns on uptake of clinical testing and research participation, the findings support calls for an immediate ban on the use of genetic test results in underwriting in Australia, and greater government oversight.

## Supplementary information

Supplementary Table S1

Supplementary file 1

Supplementary file 2
