# Supplementary material for: Genetic discrimination by Australian insurance companies: a survey of consumer experiences
Source: Eur J Hum Genet. 2019 Jul 8;28(1):108–13. doi: 10.1038/s41431-019-0426-1 (PMC6906286; doi:10.1038/s41431-019-0426-1)
Supplement: Supplementary file 1 — Supplementary Table S1 [file 41431_2019_426_MOESM1_ESM.docx]

| **Table S1: Survey results.**  CSY=colonoscopy; EY = endoscopy; AU=abdominal ultrasound; PU=pelvic ultrasound; ES=endometrial sampling; BS=breast screen; UC=urine cytology; SC=skin check; MM = mammogram; US = ultrasound PBM=prophylactic bilateral mastectomy; TH = total hysterectomy; BSO=bilateral salpingo-oopherectomy; | | | | | | | |
| --- | --- | --- | --- | --- | --- | --- | --- |
| **ID** | **Sex** | **Age** | **Personal history of cancer** | **Regular surveillance** | **Surgery** | **Experience with insurance access after genetic testing (*with explanatory free text responses if given*)** | **Was decision appealed? (*with free text responses if given*)** |
| **Lynch Syndrome mutation carriers (LSA)** | | | | | | | |
| ***High risk surveillance and some preventative surgery*** | | | | | | | |
| LS1 | F | 40 | No | annual CSY & SC;  bi-annual EY;  twice-yearly BS | TH & BSO | Increase in premium on application for life, income protection and disability insurance. “*Informed when enquiring about a new product that cost would increase because of increased risk based on new diagnosis”* | No. “*Didn't know I could appeal and was soon over diagnosis so felt overwhelmed*” |
| LS2 | F | 42 | No | annual CSY&AU;  bi-annual EY;  PU at least 3 times yearly | TH & BSO | Life, income protection, disability and mortgage insurance applications denied more than 5 times by different companies. *“When trying to obtain life insurance or switch current insurances advice given due to lynch syndrome will not cover”* | No. “*Didn't realise I could appeal. If it was too hard I would probably not bother”* |
| LS3 | F | 42 | No | annual CSY;  bi-annual EY & SC | TH & BSO | Application to increase life insurance denied | Yes. *“I challenged AMP – arguing that I now have less of a chance of developing Lynch Cancers due to preventative surgery and annual surveillance. I forwarded all the latest data and gave AMP the medical contacts – the outcome being they have now increased my insurance.”* |
| LS4 | F | 46 | No | annual CSY & EY; twice yearly BS; SC every few months | TH & BSO | Increase in premium on application for life, income protection, disability and mortgage insurance with 2 different companies, *“Just because I have Lynch but I have never had cancer”.* | No. *“I didn’t know I could”* |
| LS5 | F | 48 | No | annual CSY, AU, UC &SC;  bi-annual BC | TH & BSO | Life insurance application denied | No. *“Too difficult”* |
| LS6 | F | 50 | No | annual CSY, EY, AU, ES, UC & twice-yearly SC | PBM, TH & BSO | Life and income protection insurance application denied by 2 different companies, with no explanation provided | No. *“I have been told it is impossible to get Life Insurance once you have confirmed Lynch diagnosis”* |
| LS7 | F | 54 | No | bi-annual CSY, annual BS | TH & BSO | Life and income protection insurance application denied, with no explanation provided | No. *“I didn’t know I could appeal, and the premium became too expensive with my diagnosis”* |
| LS8 | F | 54 | No | annual CSY, EY & BS; bi-annual UC & SC | TH & BSO |  |  |
| LS9 | F | 55 | No | annual CSY, AU, PU, UC, SC; bi-annual EY; twice-yearly BS | TH & BSO | Life insurance application denied, with no explanation provided | No. *“I didn’t know there was a way to appeal. I also had a life policy that I took out prior to my Lynch diagnosis, so I just left it as is”* |
| LS10 | F | 56 | No | annual CSY, EY, AU, UC& SC; bi-annual BS | TH & BSO |  |  |
| LS11 | F | 56 | No | annual CSY, EY, AU, SC & UC; bi-annual BS | TH & BSO | *“I have decided not to apply for an increase in my Income Protection Insurance in line with my increased income because I know I will need to now disclose my Lynch Syndrome and I am unsure what that will mean in terms of premium loading”* | No. |
| LS12 | F | 57 | Skin only | annual CSY & SC, bi-annual EY & BS | TH & BSO | Life insurance application denied and increase in premium on application for income protection insurance by 2 different companies | No. *“Wasn’t considered”* |
| ***High risk surveillance, no surgery*** | | | | | | | |
| LS13 | F | 25 | No | annual CSY, EY, BS, UC, SC twice-yearly AU & PU |  | No details provided other than stating difficulty experienced with either obtaining insurance or an increased premium related to personal or family Lynch Syndrome diagnosis | - |
| LS14 | F | 26 | No | bi-annual CSY & EY |  | No details provided other than stating difficulty experienced with either obtaining insurance or an increased premium related to personal or family Lynch Syndrome diagnosis |  |
| LS15 | F | 30 | No | annual CSY; bi-annual EY |  | Life insurance application denied, with no explanation provided | No. *“Couldn’t be bothered”* |
| LS16 | F | 30 | No | annual CSY & EY |  | Increase in premium on application for life insurance, and income protection and mortgage insurance applications denied. “*I have applied for more life and income protection insurance since my diagnosis but have been refused this on medical grounds. Even though I have surveillance – apparently the insurance companies don’t take surveillance into consideration”* | Yes*. “Only by asking a question, not any formal appeal process, just gave up. My main concern is that if anything happens to me (Lynch related or not) I could lose my house”* |
| LS17 | F | 30 | No | annual CSY |  | “*Had difficulty with private health insurance”.* | Yes. No further details provided |
| LS18 | F | 37 | No | annual CSY & EY; bi-annual AU, PU & ES |  | Life, income protection, disability and mortgage insurance applications denied by 2 different companies | No. *“Didn’t think I could”* |
| LS19 | F | 37 | No | bi-annual CSY & EY |  | Life insurance application denied. *“it was declined due to my family history circumstances”* | No. *“I thought that it would be hopeless because I had a ‘cancer’ gene”* |
| LS20 | M | 38 | No | annual CSY & EY; bi-annual SC |  | Increase in premium on application for life, income protection and disability insurance | No. *“Didn’t think I could”* |
| LS21 | F | 44 | No | bi-annual CSY & EY; annual BS & UC |  | Increase in premium on application for income protection and disability insurance with 2 different companies, with no explanation provided | No. *“I didn’t think I could appeal & broker did not suggest it”.* |
| LS22 | M | 45 | No | annual CSY & EY if and when symptoms indicate |  | Life and income protection insurance applications denied. “*Just said it was classified as a cancer and I was ineligible”* | No. “*Didn't know I could*” |
| LS23 | F | 47 | No | annual CSY, EY, AU, PU, ES, UC; bi-annual BC & twice-yearly SC |  | Life, income protection and disability insurance applications denied. *“Was prohibitively very expensive by one provider and two other providers said No. One of those providers told me ‘on this occasion we cannot offer you a policy’ and no explanation provided to me”* | No. *“Did not know there was an option I assumed they can say no”* |
| LS24 | F | 51 | No | annual CSY, BS, UC; bi-annual EY & MRI |  | Life and income protection insurance application denied by 2 different companies. *“Did not qualify due to genetic disease”* | No. *“Wasn't aware there was a way to appeal”* |
| **Hereditary Breast and Ovarian Cancer mutation carriers (PH)** | | | | | | | |
| ***Some preventative surgery and/or high-risk surveillance*** | | | | | | | |
| PH1 | F | 33 | No | MM, US & MRI every 6-12 months | - | Life insurance application denied. *“Broker telling me it wouldn’t be possible”* | - |
| PH2 | F | 37 | No | US every 6-12 months  *“I bear my own costs! It’s not cheap”* | PBM, fallopian tube removal | Life, income protection, disability insurance applications denied by 2 different companies. *“Too unknown an area. Won’t cover any cancer and would proceed to load the rest.”* | Yes. *“Denied. High risk, unknown risks etc”* |
| PH3 | F | 40 | No | Bi-annual MM, US & MRI | - | Increase in premium on application for life insurance. *“I went through an insurance broker to attempt to organise life insurance and was advised that all premiums would be higher so I did not apply for it in the end”* | No. |
| PH4 | F | 40 | No | N/A | PBM, fallopian tube removal |  |  |
| ***Full preventative surgeries*** | | | | | | | |
| PH5 | F | 36 | No | N/A | PBM, BSO, TH | Application to increase life insurance cover through superannuation denied. *“I tried to change my Superannuation life insurance to a higher amount. This was denied due to my BRCA1. I had even had both of the preventative surgeries at this point. Made me feel like I had a high risk of dying and I felt extremely uneasy.”* | No. |
| PH6 | F | 47 | No | N/A | PBM, BSO | Life, income protection and disability insurance applications denied. *“Financial adviser tried to obtain insurance for me unsuccessfully because of brca1”* | Yes. *“Denied”* |
| PH7 | F | 51 | No | N/A | PBM, BSO, TH | Increase in premium on application for income protection insurance | No. |
| PH8 | F | 58 | No | N/A | PBM, BSO | Increase in premium on application for travel insurance | No. |
