# Supplementary material for: Genetic discrimination by Australian insurance companies: a survey of consumer experiences
Source: Eur J Hum Genet. 2019 Jul 8;28(1):108–13. doi: 10.1038/s41431-019-0426-1 (PMC6906286; doi:10.1038/s41431-019-0426-1)
Supplement: Supplementary file 2 — Supplementary file 1 [file 41431_2019_426_MOESM2_ESM.pdf]

## Introduction

**Life insurance, mortgage and income protection insurance are so important, yet people living with Lynch syndrome are often denied access to cover or find it impossible to extend or update cover obtained before their diagnosis.**
**Lynch Syndrome Australia are working with a team at Monash University to lobby for a change in the law around disclosing genetic results to insurance companies.**
**Your de-identifyed responses to this survey will be used to inform this change.**
**Your personal details will not be provided to any third party unless you have given us permission to do so towards the end of the survey.**

What is your name?

* How old are you?

* What is your gender?

- Female
- Male
- Prefer not to answer
- Other

* Do you live in Australia?

- Yes
- No

What is your postcode?

Which country do you currently reside?

* Have you been diagnosed with Lynch Syndrome?

- ○ Yes
- ○ No

* What gene variant was found (if known)?

- ○ MLH1
- ○ MSH2
- ○ MSH6
- ○ PMS2
- ○ Not sure

* When were you diagnosed with Lynch syndrome?

- ○ Within the last two years
- ○ Between two and five years ago
- ○ Between 5 and 10 years ago
- ○ Longer than 10 years ago

If you know the specific year, please record it here

____________________

* Have **you** had a cancer diagnosis or precancerous cells?

- ☐ No cancer diagnosis or precancerous cells
- ☐ Bowel polyps
- ☐ Colon (bowel) cancer
- ☐ Endometrial (uterine or womb) cancer
- ☐ Ovarian (ovary) cancer
- ☐ Stomach cancer
- ☐ Hepatobiliary (liver/gallbladder) cancer
- ☐ Urinary tract cancer
- ☐ Kidney cancer
- ☐ Pancreatic cancer
- ☐ Small bowel cancer
- ☐ Brain cancer
- ☐ Skin cancer (sebaceous adenoma, sebaceous epithelioma, or sebaceous carcinoma and keratoacanthoma)
- ☐ Breast cancer

Other cancer (please specify)

____________________

Have any **first degree relatives** had a cancer diagnosis or precancerous cells?

- ◯ Yes
- ◯ No
- ◯ I don't know

If yes please elaborate

[ ]

* A surveillance regime is the testing you may receive regularly to look for signs of early cancer. The surveillance regime for an individual will include those measures, such as annual colonoscopy, which are recommended for all Lynch gene carriers, as well as others that may be relevant to some individuals based on their specific family history.

Please tick one box per row to indicate the frequency of**your** surveillance regime.

Please provide a response for each line.

| | Annually | Twice a year | Three times or more a year | Once every two years | Only when symptoms indicate | Never | Not applicable |
|---|---|---|---|---|---|---|---|
| Colonoscopy | ◯ | ◯ | ◯ | ◯ | ◯ | ◯ | ◯ |
| Endoscopy (Gastroscopy) | ◯ | ◯ | ◯ | ◯ | ◯ | ◯ | ◯ |
| Abdominal Ultrasound | ◯ | ◯ | ◯ | ◯ | ◯ | ◯ | ◯ |
| Pelvic Ultrasound | ◯ | ◯ | ◯ | ◯ | ◯ | ◯ | ◯ |
| Endometrial sampling | ◯ | ◯ | ◯ | ◯ | ◯ | ◯ | ◯ |
| Breast screening | ◯ | ◯ | ◯ | ◯ | ◯ | ◯ | ◯ |
| Urine cytology | ◯ | ◯ | ◯ | ◯ | ◯ | ◯ | ◯ |
| Blood test -CEA | ◯ | ◯ | ◯ | ◯ | ◯ | ◯ | ◯ |
| Blood test - CA 19 | ◯ | ◯ | ◯ | ◯ | ◯ | ◯ | ◯ |
| Blood test - CA 125 | ◯ | ◯ | ◯ | ◯ | ◯ | ◯ | ◯ |
| Blood test PSA | ◯ | ◯ | ◯ | ◯ | ◯ | ◯ | ◯ |
| Skin check | ◯ | ◯ | ◯ | ◯ | ◯ | ◯ | ◯ |

Other (please specify)

[ ]

Please indicate which tests in your surveillance regime are prompted by past cancer experiences in your family.

- [ ] Colonoscopy
- [ ] Endoscopy (Gastroscopy)
- [ ] Abdominal Ultrasound
- [ ] Pelvic Ultrasound
- [ ] Endometrial sampling
- [ ] Breast screening
- [ ] Urine cytology
- [ ] Blood test -CEA
- [ ] Blood test - CA 19
- [ ] Blood test - CA 125
- [ ] Blood test PSA
- [ ] Skin check

Other (please specify)

* Have you ever had preventative surgery (this means surgery before any cancer in that location)?

- [ ] No
- [ ] Total hysterectomy (uterus or womb)
- [ ] Total hysterectomy and bilateral salpingo-oophrectomy (uterus or womb and ovaries)
- [ ] Colectomy (bowel)

* Do you take prophylactic aspirin?

- [ ] Yes
- [ ] No
- [ ] I am considering taking aspirin

## Page 5

* Do you currently have any of the following insurance?

| | No insurance | Insurance taken out **before** my Lynch syndrome diagnosis | Insurance taken out **after** my Lynch syndrome diagnosis |
|---|---|---|---|
| Life insurance | ☐ | ☐ | ☐ |
| Life insurance through your superannuation fund | ☐ | ☐ | ☐ |
| Income protection insurance | ☐ | ☐ | ☐ |
| Disability Insurance | ☐ | ☐ | ☐ |
| Mortgage Insurance | ☐ | ☐ | ☐ |

Other (please specify)

* Have you had any difficulty obtaining insurance or an increased premium related to you or your family's Lynch Syndrome diagnosis? [options: life insurance, income protection insurance, disability insurance, mortgage insurance, other]

- ○ Yes
- ○ No

* Describe your experiences in obtaining/maintaining the following insurances.

| | Difficulty obtaining | Increase in premium | I haven't tried to obtain this type of insurance | Have successfully obtained this type of insurance |
|---|---|---|---|---|
| Life Insurance | ○ | ○ | ○ | ○ |
| Income Protection Insurance | ○ | ○ | ○ | ○ |
| Disability Insurance | ○ | ○ | ○ | ○ |
| Mortgage insurance | ○ | ○ | ○ | ○ |

Other (please specify)

What information (if any) did your insurer provide to you to justify or explain the decision? If you have a copy of any correspondence with the insurer that you are happy to provide, please let us know

* Did you try to challenge or appeal the decision?

- Yes
- No

If yes what was the outcome of the appeal?

Page 7

* Why didn't you appeal the decision?
Was it because you didn't know there was any way to appeal the decision or was it just too confusing and hard to figure out or please expand.

## Page 8

How many times have you applied for insurance and been denied?

- ○ 1-2 times
- ○ 3-5 times
- ○ More than 5 times

Did you apply to the same insurance company or a different company?

- ○ Same company
- ○ Different company

## Page 9

* Are you willing to to discuss your survey responses with Lynch Syndrome Australia or a reputable research team working with Lynch Syndrome Australia?

○ Yes

○ No

If yes leave your phone number and/or email address

* Are you willing to be contacted regarding speaking with the media about your experiences?

○ Yes

○ No

If yes leave your phone number and/or email address
Write as above if you have already left these details in the previous question.

* Would you be interested in participating in future surveys and consultation to support Lynch Syndrome Australia's advocacy work so we can ensure the unique needs and concerns of families with Lynch syndrome are recognised, understood and given due consideration?

- Yes
- No

If yes leave your phone number and/or email address
Write as above if you have already left these details in the previous question.
