# Supplementary material for: Genetic discrimination by Australian insurance companies: a survey of consumer experiences
Source: Eur J Hum Genet. 2019 Jul 8;28(1):108–13. doi: 10.1038/s41431-019-0426-1 (PMC6906286; doi:10.1038/s41431-019-0426-1)
Supplement: Supplementary file 3 — Supplementary file 2 [file 41431_2019_426_MOESM3_ESM.pdf]

Genetic testing and insurance - Pink Hope survey 2

1. Name

2. Year of birth

3. Gender

- male
- female
- Other (please specify)

4. Do you live in Australia?

- yes
- no

5. What is your postcode?

6. Have you or a first degree relative had a positive genetic test for a gene mutation that predisposes you or the relative to breast and/or ovarian cancer?
(note that this includes genetic testing done in a research context)

- yes, I have had a positive genetic test
- yes, my relative has had a positive genetic test
- no

7. Have you made a decision to NOT have a genetic test for a known family mutation?

- ◯ yes
- ◯ no

8. Can you elaborate further/provide reasons for this decision?

9. For which gene have you or your family member had a positive genetic test?

- ◯ BRCA1
- ◯ BRCA2
- ◯ PALB2
- ◯ ATM
- ◯ Other
- ◯ Unsure

10. Have you had one or more diagnoses of breast, ovarian and/or other cancer?

- ☐ yes, breast cancer
- ☐ yes, ovarian cancer
- ☐ no
- ☐ Other (please specify)

11. What year was each cancer diagnosed?

12. Have any of your first degree relatives had one or more diagnoses of breast and/or ovarian cancer?

- [ ] yes, breast cancer
- [ ] yes, ovarian cancer
- [ ] no
- [ ] Other (please specify)

13. Have you had preventative surgery associated with your genetic test result?

- [ ] Unilateral mastectomy
- [ ] Bilateral mastectomy
- [ ] Removal of ovaries and fallopian tubes
- [ ] Hysterectomy
- [ ] No surgery
- [ ] Other (please specify)

14. Do you have a screening programme of regular breast imaging (mammogram, MRI and/or ultrasound)?

- ( ) yes
- ( ) no

15. What is your screening programme?

	6 monthly or more frequently	Every 6-12 months	Every 12-18 months	Every 2 years	Less than every 2 years	Never
Mammogram	◯	◯	◯	◯	◯	◯
MRI	◯	◯	◯	◯	◯	◯
Ultrasound	◯	◯	◯	◯	◯	◯

Other (please specify)

16. Do you have any further comments to make regarding your screening programme?

17. Do you currently have any of the following insurance?

	no insurance	insurance taken out before positive genetic test	insurance taken out after positive genetic test
Life insurance	◯	◯	◯
Life insurance through superannuation fund	◯	◯	◯
Income protection insurance	◯	◯	◯
Disability insurance	◯	◯	◯
Mortgage insurance	◯	◯	◯

18. Have you had any difficulty obtaining insurance or an increased premium related to your positive gene test?

	insurance cover denied	increase in premium (loading)	I haven't tried to obtain this type of insurance	I have successfully obtained this type of insurance after my genetic testing without any loading	I already had this type of insurance before my genetic testing
Life insurance	◯	◯	◯	◯	◯
Life insurance through superannuation fund	◯	◯	◯	◯	◯
Income protection insurance	◯	◯	◯	◯	◯
Disability insurance	◯	◯	◯	◯	◯
Mortgage insurance	◯	◯	◯	◯	◯
Travel insurance	◯	◯	◯	◯	◯

Other (please specify)

19. What information (if any) did the insurer/s provide to you to justify or explain the decision? If you remember the name of the insurer/s and/or have copies of any correspondence with the insurer/s that you are happy to provide, please let us know.

20. Did you try to challenge or appeal the insurer's decision?

◯ yes

◯ no

21. What was the outcome of the appeal?

22. If not, why not?

23. How many times have you applied for and been denied insurance following your positive gene test?

- Never
- 1-2 times
- 3-5 times
- More than 5 times

24. Did you apply to the same insurance company or different companies?

- ◯ Same insurance company
- ◯ Different companies

25. Are you willing to discuss your survey responses with Pink Hope or a reputable research team working with Pink Hope?

- ◯ yes
- ◯ no

26. Please provide your best contact phone number and/or email address

27. Are you willing to be contacted by Pink Hope regarding the possibility of speaking with the media about your experiences?

- ○ yes
- ○ no

28. Please provide your best contact phone number and/or email address (or write "as above" if you have already provided these details in the previous question)

29. Do you have any further comments to make about this issue?
